# Prevalence of Cytomegalovirus in Patients With Newly Diagnosed, Flare-Up, and Acute Severe Ulcerative Colitis

**DOI:** 10.7759/cureus.99449

**Published:** 2025-12-17

**Authors:** Milan Shrestha, Ramila Shrestha, Ajit Khanal, Bhupendra Basnet, Nandu S Poudyal, Ajay K Yadav, Mukesh S Paudel

**Affiliations:** 1 Gastroenterology, Bir Hospital, Kathmandu, NPL; 2 Gastroenterology, Bir hospital, Kathmandu, NPL

**Keywords:** acute severe ulcerative colitis (asuc), cytomegalovirus (cmv), eosin and hematoxylin stains, immunohistochemical stains (ihc), inflammatory bowel disease (ibd)

## Abstract

Background: Cytomegalovirus (CMV) is a ubiquitous virus belonging to the human herpesvirus that can reactivate in immunosuppressed ulcerative colitis (UC) patients, leading to frequent relapses and acute severe colitis. In inflammatory bowel disease, CMV colitis is linked to higher mortality, morbidity, and healthcare costs. CMV immunohistochemistry (IHC) is far more sensitive than routine H&E staining and should be part of the evaluation of UC patients with severe exacerbations or steroid-refractory disease during standard-of-care medical or surgical treatment. Severe CMV infection can cause fulminant colitis, toxic megacolon, or perforation, requiring urgent surgery. Colonoscopic findings may include patchy erythema, exudates, edema, erosions, deep ulcers, and pseudo-tumors. Despite growing evidence of CMV reactivation in UC, no official prevalence data exist for Nepal.

Objectives: Primary aim was to determine the prevalence of cytomegalovirus infection in newly diagnosed, flare-up and acute severe UC, and secondary aim was to evaluate the colonoscopic findings in patients with cytomegalovirus infection superimposed with UC

Materials and Methods: A cross-sectional observational study was conducted at a tertiary center in the central part of Nepal from August 2024 to August 2025, among a group of 39 UC patients who were either newly diagnosed, UC in flare, or patients presenting with acute severe UC. All patients underwent either a flexible sigmoidoscopy or colonoscopy, and biopsies were taken from the inflamed mucosa for evaluation using histopathology with H&E stain and immunohistochemistry (IHC) with the CCH2 and DDG9 antibodies as markers for CMV. Data on demographic and clinical characteristics, endoscopic grading with Mayo’s severity score, and extent of disease, along with stool examination for infectious etiology, including Clostridioides difficile along with relevant history for possible risk factors for relapse, such as complementary drug use, recent antibiotic and nonsteroidal anti-inflammatory drugs (NSAIDs) use, and non-compliance to therapy along with history of recent travel before onset of symptoms were obtained. Statistical analysis was done using IBM SPSS Statistics for Windows, version 21 (IBM Corp, Armonk, NY, USA).

Results: The prevalence of cytomegalovirus infection in ulcerative colitis was found in 4 of 39 UC patients (10.25%). It was most common in acute severe cases (2/7, 28.57%) and in flares (2/26, 7.69%), with no cases in newly diagnosed patients (0/6). Immunohistochemistry detected all four CMV colitis cases, while histopathology identified only one, indicating that IHC is more accurate than histology alone for diagnosis.

Conclusion: This study suggests cytomegalovirus infection as an important risk factor for relapse in patients with UC and should be evaluated with immunohistochemistry along with a histopathological study.

## Introduction

Ulcerative colitis (UC) is characterized by chronic inflammation of the large intestine involving the rectum with progressive proximal involvement that involves the additional areas of colon [1]. Patients with UC are frequently on immunosuppressants. This, in the background of chronic inflammation in the host, can lead to frequent relapses and acute severe colitis due to cytomegalovirus (CMV) reactivation. Goodpasture and Talbert in 1921 suggested the term “cytomegalia” and proposed that it be attributed to viral etiology [2]. The first case of CMV infection in UC was described by Powell in 1961. Ribbert first demonstrated inclusion-bearing cells in 1881 [3]. The coexistence of CMV in inflammatory bowel disease (IBD) colitis is associated with high rates of colectomy and morbidity risks of hospitalization, inpatient length of stay, and increased mortality in those with colitis [4]. CMV, a member of the Herpesviridae family, is a highly prevalent viral infection, with an estimated 40-100% of adults infected by the age of 40. In immunocompetent individuals, primary CMV infection is typically asymptomatic, though it may present with mild manifestations such as fever, myalgia, or lymphadenopathy. Similar to other herpesviruses, CMV establishes lifelong latency within the host and has the potential to reactivate, particularly in individuals with compromised immune function [5]. CMV colitis superimposed with UC may present as mild to fulminant colitis. In cases of severe fulminant infection, CMV may lead to life-threatening complications such as fulminant colitis, toxic megacolon, and bowel perforation, often requiring urgent surgical intervention. Factors associated with CMV reactivation in patients with UC include female sex, pancolitis, age greater than 30 years, use of immunosuppressive therapy, disease duration of less than 60 months, and a blood leukocyte count below 11/nL [6].

The diagnosis of CMV in UC requires a variety of diagnostic modalities, such as serology for CMV, colonoscopy with biopsy for histopathology, IHC, and/ or CMV polymerase chain reaction (PCR) from the tissue [7]. Colonoscopy findings in CMV colitis include patchy erythema, exudates, microerosions, diffusely edematous mucosa, multiple mucosal erosions, deep ulcers, and pseudo-tumor [8]. CMV DNA was only detectable in segments of inflamed mucosa and not in normal tissue of the same patient, suggesting that colonic biopsies should be obtained from areas of active inflammation [9]. Furthermore, tissue from the base and edges of ulcers was found to have the highest densities of CMV-positive cells, and biopsies from the left colon identified the majority of patients with UC with CMV [10,11]. The characteristic histopathological appearance of cytomegalic cells includes a basophilic intranuclear inclusion, sometimes surrounded by a clear halo of cytoplasm, resembling an "owl's eye". In most cases, inclusion bodies are abundant and easily seen on H&E staining, but due to the potential high false-negative rate, IHC is recommended to improve the diagnostic yield [12].

## Materials and methods

Study design and setting

This was a hospital-based cross-sectional study conducted at the Department of Gastroenterology, National Academy of Medical Sciences (NAMS), Bir Hospital, Kathmandu, Nepal, from August 2024 to August 2025. Bir Hospital is a tertiary-level hospital located in the capital city of Nepal, where patients from across the country visit for specialty care.

Study participants

All adult patients (> 18 years) with newly diagnosed, flare-up, and patients admitted for acute severe UC who consented to colonoscopy and biopsy were included in the study. Patients with ulcerative colitis in remission and a previous diagnosis of cytomegalovirus colitis among UC patients, either treated or untreated in the past, were excluded from the study.

Sample size

The sample size calculation is done by the ‘pwr’ package in R (R core team, Vienna, Austria, 2023). The sample size for each group in a two-sample proportion is given by

Sample size:

N= Z^2^pq/d^2^ where

N = desired sample size

Z = standard deviation, usually set at 1.96, which corresponds to the 95 % confidence interval

P = proportion in a target population [A1] 

q = proportion in a target population not having the particular characteristics

d = degree of accuracy required

In my case, 

N= (1.96)^2^ x 7 x 93 /64 equals to 39.07

Sample size obtained = 39.

Yadegarynia et al. determined the prevalence of CMV and found that 7% and hence the value of “P” was taken as 7 [13].

Procedure details

All the consecutive patients undergoing workup for new onset UC or flare of UC in the Department of Gastroenterology, NAMS, Bir Hospital, on both inpatient and outpatient basis were evaluated with a detailed history like urgency, per-rectal bleeding, increased frequency of bowel movements, mucus discharge, and tenesmus. A thorough clinical examination was performed to assess hemodynamic stability, severity of the disease, and presence of other underlying co-morbidities. Hematological examination, including complete blood count, erythrocyte sedimentation rate( ESR), and Biochemical tests like C-reactive protein (CRP), and microscopic examination for routine stool examination and stool for Clostridioides Toxin A, Toxin B, and Glutamate dehydrogenase, along with imaging studies, was performed. The patients with UC were evaluated with a colonoscopy. They were performed by the faculty or residents under supervision, and their mucosal lesions were assessed. The biopsy was taken from the inflamed mucosa for HPE and IHC as per ACG guidelines [1]. We sent the biopsy form, an inflamed mucosa predominantly left-sided biopsy, and biopsies were taken from the edge as well as the base of the ulcer. A minimum of six biopsies were taken from the inflamed mucosa. A sample jar containing 6-8 tissue samples was placed in a formalin solution was sent to the pathological evaluation after proper tagging. Histopathological evaluation was done with H&E stain, and CMV inclusion bodies were looked for in the H&E stain. For IHC, the samples were sent in formalin, and the cytomegalovirus tissue antigens were subjected to specific antibodies (CCH2 and DDG9). This produces brownish to blackish stains in the nuclei and suggests tissue invasion.

Acute severe ulcerative colitis (ASUC) was defined by the modified Truelove and Wits criteria that combine the presence of bloody stools ≥6 times a day with features of systemic toxicity such as temperature ≥37.8°C, hemoglobin <10.5 g/dL, erythrocyte sedimentation rate <30 mm/h, and/or a pulse rate of ≥90 bpm [14].

The extent of the disease was defined according to the currently used Montreal classification as proctitis (E1, usually defined as ≤15 cm of inflammation), left-sided colitis (E2, defined as more than proctitis, but the extent stops at or distal to the splenic flexure), or extensive colitis (E3, defined as extension proximal to the splenic flexure, with pancolitis defining the entire rectum and colon including the cecum) [1].

Flare in UC was determined as per the re-emergence of symptoms along with Mayo's endoscopic subscore > 1 from the remission state.

Statistical analysis

A structured proforma was used to collect data. Statistical analysis was done using IBM SPSS Statistics for Windows, version 21 (IBM Corp., Armonk, NY, USA). Descriptive analysis was conducted using means (and standard deviations) or medians (and interquartile ranges), as appropriate, along with proportions. The prevalence of CMV in patients with UC was obtained as the proportion of UC patients with histological evidence of cytomegalovirus or immunohistochemical diagnosis.

## Results

Among 39 consecutive patients who presented with UC, the mean age of the participants was 41.46 with a standard deviation of 14.95, with a range of participants from 22- 70 years, with predominant participants in the age group of 30-39 years. No significant gender differences were present in the study population. The majority (30/76.92 %) of patients were non-smokers. The mean body mass index of the study population was 20.04±1.68 kg/m². Most of the participants presented with flare of UC 26 (66.67 %), followed by acute severe ulcerative colitis 7 (17.94%) and newly diagnosed cases 6 (15.38%) each. Most patients had left-sided colitis (E2) followed by extensive colitis (E3) and proctitis (E1) disease in frequency of 22 (56.4%), 14 (35.9%), and 3 (7.7%) each. Mayo’s endoscopic subscore in the study population was M2 disease (21/53.8%), followed by M3 (14/35.9%) and M1(4/10.3%) each (Table 1).

**Table 1:** Sociodemographic data and clinical characteristics of ulcerative colitis

Age	Mean Age	41.46 with SD 14.95	Range (20–77 Years)
	20–29 years	6	17.2
	30–39 years	14	31.0
	40–49 years	11	27.6
	50–59 years	2	3.4
	60–69 years	3	10.3
	70–79 years	3	10.3
Sex	Male	21	53.8
	Female	18	46.2
	Body mass index	20.04± 1.68 kg/m²	Range -168-16.2 kg/m^2^
Smoking status	Smoker	3	7.69
	Ex-smoker	6	15.4%
	Non-smoker	30	76.92
Extent	E1	3	7.7
	E2	22	56.4
	E3	14	35.9
Mayo severity subscore	M1	4	10.3
	M2	21	53.8
	M3	14	35.9
Type of UC	Flare-up	26	66.67
	ASUC	7	17.94
	Newly diagnosed	6	15.38

**Figure 1:**
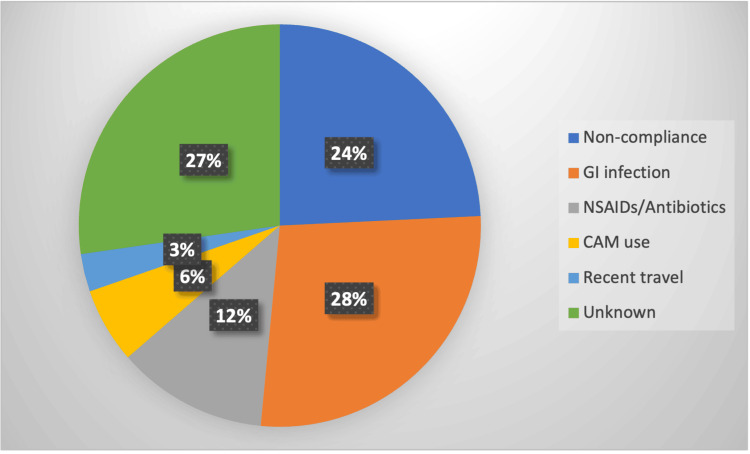
Etiology of relapse in ulcerative colitis

Among the 33 of 39 patients with flare or acute severe UC (excluding newly diagnosed cases), the cause could not be identified. The remaining cases were attributed to gastrointestinal infections, including *Clostridioides difficile* infection (4/33), followed by non-compliance, recent NSAIDs or antibiotic use within the past two weeks, use of complementary medicines, and recent travel history (Figure 1).

**Figure 2:**
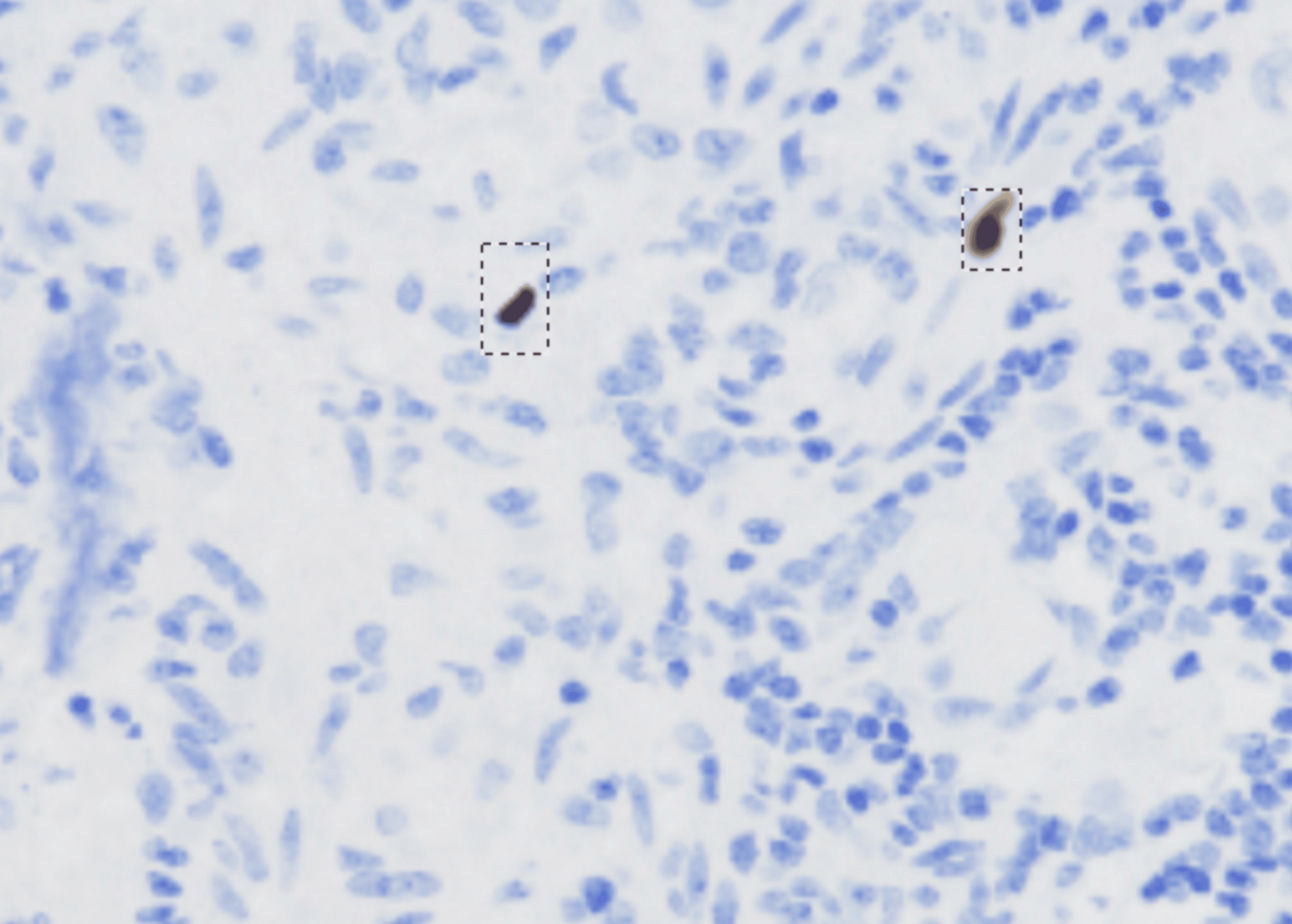
Positive IHC study for CMV with brownish black stain on IHC IHC: immunohistochemistry; CMV: cytomegalovirus

**Figure 3:**
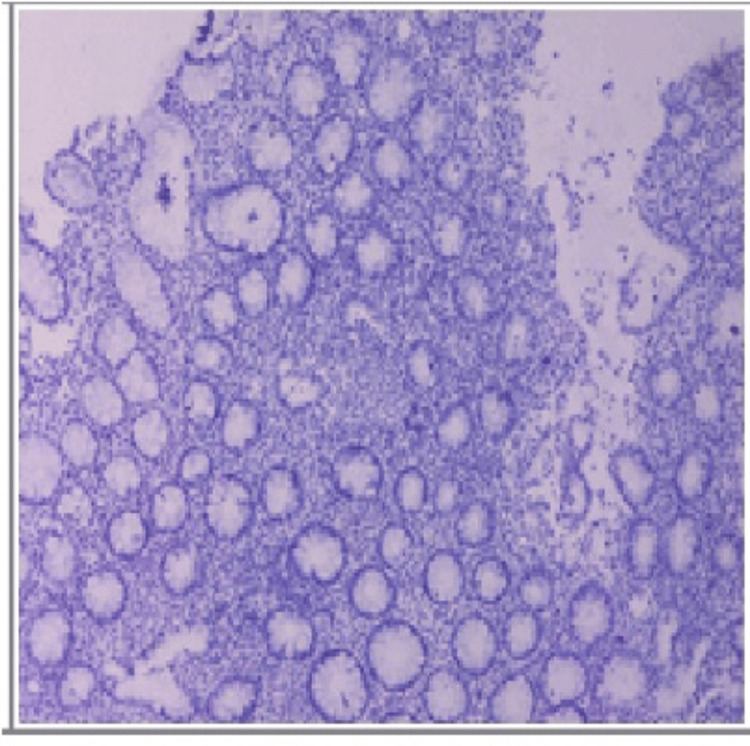
IHC with a negative result IHC: immunohistochemistry

Four patients out of 39 participants with UC tested positive for CMV, with one participant among them demonstrating the histopathological findings of cytomegalovirus. All four patients showed positive staining in the IHC study for the CCH2 and DDG9 antibodies, with representative positive and negative IHC results of one patient from both positive and negative findings depicted in the figure below. Participants who showed features of cytomegalovirus on histopathology also tested positive on IHC (Figures 2-3).

## Discussion

The prevalence of CMV colitis in UC was estimated to be 10.28 %, with 28.57 % in ASUC and 7.69 % in flare-up of ulcerative colitis. To the best of our knowledge, this is the first study from Nepal to evaluate the hospital-based prevalence of cytomegalovirus infection among patients with UC. Our study correlates with Yadegarynia et al., who found the overall prevalence of CMV in UC to be 7% (6 out of 86 patients). In the same study, 30% (2 out of 6) required colectomy, compared to 7.5% (6 out of 80) of the CMV-negative cases, highlighting the serious prognosis for CMV-positive cases in UC [13]. According to Takahashi et al., the reported prevalence of CMV infection in ASUC ranges from 21% to 34%, which is consistent with the findings of our study. [15]. IHC was found to be superior to histopathology in diagnosing CMV colitis in UC, which correlates with a previous study by Kim et al. In the study, only one patient out of 55 patients tested positive with histopathology, with IHC determining the rest of the cases [16]. A study of 114 subjects by Domènech et al. with various forms of UC found CMV disease only in six steroid-refractory UC patients. CMV disease was diagnosed in five out of the six patients after 7-10 days on cyclosporine following immunosuppression, and the complication rates are high with CMV reactivation if not detected early [17]. In a study by Bhatia et al. with 58 patients in UC, 12 patients tested positive for CMV. Two-thirds of patients (8/12) had severe disease, while the remaining (4/12) had moderate disease activity. This study showed an increase in colectomy rates as well as severe disease in patients with UC and CMV, which is consistent with the findings of our study. [18].

CMV may reactivate due to inflammation in UC and immunosuppressive treatment, such as malnutrition or dysfunction of the defense factors. Inflammation and mucosal damage in the bowel mucosa led to the secretion of pro-inflammatory cytokines, leading to chemotaxis of CMV-infected macrophages to the damaged mucosa. Progressive inflammation and vasculitis lead to severe ischemia and transmural necrosis, resulting in increased mucosal erosion and deep ulcers. In contrast, CMV has pro-inflammatory effects promoting the expression of cytokines such as tumor necrosis factor-alpha (TNF-α), interferon-γ, interleukins (IL-2 and IL-8), chemokines, and cellular adhesion molecules [19]. Colonoscopy findings in CMV colitis are not specific and may include patchy erythema, exudates, micro erosions, diffusely edematous mucosa, multiple mucosal erosions, deep ulcers, and pseudo-tumor. This usually presents as an exacerbation of underlying UC with moderate to severe endoscopic subcore in most cases [8]. ACG guidelines 2025 now recommend biopsy of the involved mucosa for assessment of CMV in all moderate to severe UC and ASUC due to the high morbidity and mortality in the UC patients with CMV [1].

Limitations of the study

This was a single-center study and has several limitations. Firstly, we did not categorize UC into steroid-responsive and steroid-refractory, limiting the diagnostic values of the study. Secondly, pathologists were not informed of the study, limiting the diagnostic efficacy of the routine histopathological study. Thirdly, we did not follow up with the patients with CMV after discharge due to the cross-sectional nature of the study. Hence, we could not evaluate the prognosis of the patients after antiviral therapy. Fourthly, the sample size evaluated was small, providing restricted information.

Recommendations

We recommend a large multi-center randomized controlled trial for further confirmation of the findings. We also recommend the UC patient’s medication history for risk of reactivation, as per the drug use.

## Conclusions

This study highlights a notable prevalence of cytomegalovirus infection among patients presenting with ulcerative colitis who present with relapse of the disease. These findings underscore the value of routine biopsy for CMV in UC during flare and ASUC. The findings suggest the consideration of CMV in UC as a diagnostic possibility in patients with moderate to severe endoscopic findings. This study also highlights the fact that IHC should always be done routinely along with histopathology to increase the yield of diagnosis.

## References

[1] Rubin DT, Ananthakrishnan AN, Siegel CA, Barnes EL, Long MD (2025). ACG Clinical Guideline Update: Ulcerative colitis in adults. Am J Gastroenterol.

[2] Riley HD Jr (1997). History of the cytomegalovirus. South Med J.

[3] Powell RD, Warner NE, Levine RS, Kirsner JB (1961). Cytomegalic inclusion disease and ulcerative colitis: Report of a case in a young adult. Am J Med.

[4] Kwon J, Fluxá D, Farraye FA, Kröner PT (2022). Cytomegalovirus-related colitis in patients with inflammatory bowel disease. Int J Colorectal Dis.

[5] Maher MM, Nassar MI (2009). Acute cytomegalovirus infection is a risk factor in refractory and complicated inflammatory bowel disease. Dig Dis Sci.

[6] Maresca R, Varca S, Di Vincenzo F (2023). Cytomegalovirus infection: An underrated target in inflammatory bowel disease treatment. J Clin Med.

[7] Boivin G, Handfield J, Toma E (1998). Evaluation of the AMPLICOR cytomegalovirus test with specimens from human immunodeficiency virus-infected subjects. J Clin Microbiol.

[8] Ljungman P, Griffiths P, Paya C (2002). Definitions of cytomegalovirus infection and disease in transplant recipients. Clin Infect Dis.

[9] Roblin X, Pillet S, Oussalah A (2011). Cytomegalovirus load in inflamed intestinal tissue is predictive of resistance to immunosuppressive therapy in ulcerative colitis. Am J Gastroenterol.

[10] Zidar N, Ferkolj I, Tepeš K, Štabuc B, Kojc N, Uršič T, Petrovec M (2015). Diagnosing cytomegalovirus in patients with inflammatory bowel disease--by immunohistochemistry or polymerase chain reaction?. Virchows Arch.

[11] McCurdy JD, Enders FT, Jones A, Killian JM, Loftus EV Jr, Bruining DH, Smyrk TC (2015). Detection of cytomegalovirus in patients with inflammatory bowel disease: Where to biopsy and how many biopsies?. Inflammatory Bowel Diseases.

[12] Cotte L, Drouet E, Bissuel F, Denoyel GA, Trepo C (1993). Diagnostic value of amplification of human cytomegalovirus DNA from gastrointestinal biopsies from human immunodeficiency virus-infected patients. J Clin Microbiol.

[13] Yadegarynia D, Tehrani S, Roohi M, Gachkar L, Nadji SA, Hashemi M, Molanaei S (2018). Prevalence of cytomegalovirus infection in patients with ulcerative colitis: A prospective cross-sectional study in Tehran, Iran. Iran J Microbiol.

[14] Truelove SC, Witts LJ (1955). Cortisone in ulcerative colitis; final report on a therapeutic trial. Br Med J.

[15] Takahashi Y, Tange T (2004). Prevalence of cytomegalovirus infection in inflammatory bowel disease patients. Dis Colon Rectum.

[16] Kim JJ, Simpson N, Klipfel N, Debose R, Barr N, Laine L (2010). Cytomegalovirus infection in patients with active inflammatory bowel disease. Dig Dis Sci.

[17] Domènech E, Vega R, Ojanguren I (2008). Cytomegalovirus infection in ulcerative colitis: A prospective, comparative study on prevalence and diagnostic strategy. Inflamm Bowel Dis.

[18] Bhatia Bhatia, Jena Sumit, Kudva A (2023). Cytomegalovirus infection in ulcerative colitis: An Ambispective Study from a single center. J Gastro Infect.

[19] Esen S, Saglik I, Dolar E (2024). Diagnostic utility of cytomegalovirus (CMV) DNA quantitation in ulcerative colitis. Viruses.

